# Individualization of the FSH starting dose in IVF/ICSI cycles using the antral follicle count

**DOI:** 10.1186/1757-2215-6-11

**Published:** 2013-02-06

**Authors:** Antonio La Marca, Valentina Grisendi, Simone Giulini, Cindy Argento, Alessandra Tirelli, Giulia Dondi, Enrico Papaleo, Annibale Volpe

**Affiliations:** 1 Mother-Infant Department, Institute of Obstetrics and Gynecology, University of Modena and Reggio Emilia, 41100, Modena, Italy; 2 Centro Natalità, Gynecological-Obstetrics Department, San Raffaele Hospital, Vita-Salute San Raffaele, Milano, Italy

**Keywords:** Starting FSH dose, ART, AFC, FSH, Ovarian reserve

## Abstract

**Background:**

The FSH starting dose is usually chosen according to women’s age, anamnesis, clinical criteria and markers of ovarian reserve. Currently used markers include antral follicle count (AFC), which is considered to have a very high performance in predicting ovarian response to FSH. The objective of the present study to elaborate a nomogram based on AFC for the calculation of the appropriate FSH starting dose in IVF cycles.

**Methods:**

This is a retrospective study performed at the Mother-Infant Department of Modena University Hospital. IVF patients (n=505) were subjected to blood sampling and transvaginal ultrasound for measurement of serum day3 FSH, estradiol and AFC. The variables predictive of the number of retrieved oocytes were assessed by backwards stepwise multiple regression. The variables reaching the statistical significance were then used in the calculation for the final predictive model.

**Results:**

A model based on age, AFC and FSH was able to accurately predict the ovarian sensitivity and accounted for 30% of the variability of ovarian response to FSH. An FSH dosage nomogram was constructed and overall it predicts a starting dose lower than 225 IU in 50.2% and 18.1% of patients younger and older than 35 years, respectively.

**Conclusions:**

The daily FSH dose may be calculated on the basis of age and two markers of ovarian reserve, namely AFC and FSH, with the last two variables being the most significant predictors. The nomogram seems easily applicable during the daily clinical practice.

## Background

The main aspect of the “individualization” in IVF cycles is to offer every single patient the best therapy tailored on her own characteristics thus allowing a high chance of success and of course minimizing the risks deriving from ovarian stimulation. Choosing different doses of gonadotrophins for different patients is the most important clinical decision in the personalization of therapy.

Although exogenous FSH has been used for decades, and millions of cycles have been performed worldwide, criteria to select the proper starting FSH dose have not yet been completely identified.

Usually clinicians choose the FSH starting dose according to anamnesis and clinical criteria, the most important being the outcome of previous IVF cycles. If no previous cycles have been performed, the choice will be based on criteria as women's age, BMI and markers of ovarian reserve [1,2].

Currently used markers of ovarian reserve include FSH, AMH and antral follicle count (AFC), with the last two markers having the best performance in predicting ovarian response to exogenous FSH [3-5]. Ovarian antral follicles larger than 2 mm are extremely sensitive and responsive to FSH and are defined as "recruitable". They can be visualized and measured with transvaginal ultrasound and the total number of 2–10 mm follicles in both the ovaries represents the AFC [6,7]. Hence the AFC estimates with a very good accuracy the extent of the pool of follicles on which the exogenous FSH will act.

Several studies have been published reporting a very good correlation between AFC and ovarian response in IVF programs [8,9]. AFC is also correlated to the onset of menopausal transition [10] thus indicating the close relationship with the quantitative aspect of ovarian reserve.

AMH and AFC are nowadays considered two markers with similar diagnostic performance [4,8]. Indeed AMH and AFC actually measure the same thing: the pool of recruitable follicles. While AMH appears to have important advantages over AFC, as the lowest intra- and inter-menstrual cycle variability and the operator-independence, the great advantage of the AFC is the possibility of its measurement at the same moment in which clinicians examine the patient; hence, with great certainty we can assert that AFC will continue to be a widely used marker of ovarian reserve.

Although tailored therapy based on markers of ovarian reserve appears to be an agreed upon approach by most, studies suggesting how to determine individualized therapy compared to ‘one size fits all’ are scarce. On the use and efficacy of the single value of AMH in tailored treatment, two studies have been published [11,12]. In both studies the FSH starting dose decreased with increasing basal serum AMH and both studies indicated that the treatment strategy based on AMH may lead to a reduction of both excessive responses and cancelled cycles [11,12]. Recently a more complete and easy to use nomogram has been elaborated in order to calculate the most appropriate FSH starting dose in IVF cycles [13]. The nomogram is based on patient’s age, serum day3 FSH and AMH and may be the basis for the individualization of the FSH dose for all patients in those centers using AMH to investigate ovarian reserve.

While an important role for AFC in the identification of the extremes of ovarian response has been proven [8,9], its role in the individualization of the therapeutic strategy such as the choice of the FSH starting dose still need to be clarified. For this reason, we wished to investigate whether it is possible to elaborate a nomogram based on patients characteristics and AFC, able to suggest the appropriate starting dose of exogenous gonadotrophin in IVF cycles, aiming to possibly reduce the extremes of ovarian response.

## Methods

We retrospectively analyzed the database containing clinical and laboratory information on IVF treatment cycles carried out at the Mother-Infant Department of University Hospital since the introduction of the routinely use and registration of antral follicle count. These data have been collected and recorded in the registered database in our fertility centre in Modena, Italy. Cycles were selected for analysis if all the following inclusion criteria were satisfied: 1. first IVF/ICSI attempt, 2. regular menstrual cycle (25–35 days), 3. female age ≤ 40, 4. treatment with a long GnRH agonist protocol, 5. starting FSH dose of 225 IU per day for the first 5–6 days, 6. complete patient records on anamnestic, clinical, IVF cycle characteristics, 7. serum day3 FSH, estradiol and day3 AFC measured not more than 3 months before the IVF cycle. All patients had been trying to conceive for at least 12 months and had undergone a fertility workup. Clinical exclusion criteria were: irregular cycles, evidence of PCO status, previous ovarian surgery, endometriosis, basal day3 FSH >15IU/L, presence of ovarian cysts, history of PID, use of hormonal contraception in the previous 3 months, any known metabolic or endocrinological disease.

Of the eligible patients five hundred and five were selected while more than fifteen hundreds women were excluded on the basis of inclusion and exclusion criteria.

The long GnRH agonist protocol was based on the administration of daily leuprorelin (Enantone die, Takeda, Italy) on day 21 of the previous luteal phase of the stimulation cycle. Recombinant FSH at a dose of 225 IU/day (Gonal F, Merck Serono, Italy) subcutaneously was commenced when pituitary desensitization was achieved (~14 days after the initiation of GnRH agonists), as evidenced by the absence of ovarian follicles >10 mm and endometrial thickness <4 mm on transvaginal ultrasound examination, and then the dose was adjusted on day 6–7 of stimulation according to the ovarian response. When at least three follicles reached ≥ 18 mm, 10000 IU of hCG (Gonasi, IBSA, Italy) were administrated intramuscularly and 34–36 hours later follicles were aspirated under patient sedation. Cycle cancellation occurred because of absent response when no follicle growth was evident following at least one week of FSH administration. Cycle cancellation for excessive response was applied in the occurrence of ≥20 follicles (total number) ≥11 mm in mean diameter and/or E_2_ level ≥4,000 pg/mL on the day on which final oocyte maturation induction was scheduled. All patients gave written informed consent at the time of the IVF cycle for both the procedure and for recording and using laboratory and clinical data related to their medical history for clinical research purpose.

### Ultrasound and hormone assay

Blood samples were taken between 8:00 a.m and 12:00 p.m. from the cubital vein, in the early follicular phase (day 3) prior to any IVF-related drug administration. Serum FSH was measured by a chemiluminescent assay (ADVIA Centaur, Siemens Healthcare Diagnostics, Italy). The sensitivity of the assay was 0.1IU/L; intra- and inter-assay coefficients of variation were 2.7 and 3.1%, respectively. Estradiol was measured by a competitive chemiluminescent assay (ADVIA Centaur Siemenes Healthcare Diagnostics, Italy). The sensitivity was 10 pg/ml and the intra- and inter-assay coefficients of variations were 3.1% and 5%, respectively.

All ultrasound examinations were carried out in the same day of blood sample by using the 6.5 MHz vaginal probe on a Esaote AU4 Idea (Esaote, Milan, Italy). Examination of the ovary was established by scanning from the outer to the inner margin. All 2–10 mm follicles in site were counted in each ovary. Follicular size is measured using the internal diameters of the area. The mean of two perpendicular measurements was assumed to be the follicular size. The sum of counts in both ovaries produced the AFC.

### Statistical analyses

Cycles cancelled for absent response were assigned a number of retrieved oocytes of zero. Cycles cancelled for excessive response were arbitrarily assigned a number of retrieved oocytes corresponding to the 90° centile of the number of retrieved oocytes in the overall population (90° centile=18 oocytes). The variables predictive of the number of retrieved oocytes were assessed by backwards stepwise multiple regression. Backward selection of parameters was applied, using Wald P<0.05 for entry and P>0.1 for removal. The variables reaching the statistical significance in multivariate regression analysis were then used in the calculation for the final model elaborated in order to determine the dose of gonadotropin needed to achieved the desired response. Regression coefficients for each variables were incorporated in an algorithm were the dependent variable was the dose of FSH needed to obtain the desired response. In this study the desired response was the median number of oocytes retrieved in the overall female population (nine oocytes). As already reported elsewhere [13] the increase in the number of cycles with an oocyte yield close to the median value may be followed by a reduction in the number of cycles with an extreme ovarian response. Statistical analysis was performed using the software Stata 10 (StataCorp, Texas, USA).

## Results

In total 505 started cycles were available for statistical analysis. Patients and IVF cycles characteristics are reported in Table 1. Mean (±SD) age of patients was 34.1 (±3.9) yrs (range: 22–40). The 92.6% (n=468) of women reached the oocyte pick-up. Sixteen cycles were cancelled because of absent follicle growth and 21 were cancelled because of excessive ovarian response. Mean (±SD) number of retrieved oocytes was 9.2 (±6.4).

**Table 1:** Patients and IVF cycles characteristics

**Variables**	**n=505**
Age, years (M±SD)	34.1±3.9
BMI (kg/m2)	21.86±2.7
Smokers (%)	17
Non Smokers (%)	83
Duration of infertility (months)	28.4±27
Type of infertility	
Primary (%)	72
Secondary (%)	28
Causes of infertility ^a^	
Male factor	38.7
Tubal factor	12.3
Unexplained	52
Total day3 AFC (M±SD)	9.6±5.8
day 3 FSH, IU/L ( M±SD)	6.3±3.4
Duration of stimulation, days (M±SD)	11.1±2.1
Total FSH administered, IU (M±SD)	2500±1050
Number of retrieved oocytes per patient^b^, (M±SD; median)	9.2±6.4; 8.7

^a^ some couples had more than one cause of infertility.

^b^ patients reaching pick up=468. 16 patients were cancelled because of absent response, 21 patients were cancelled because of high risk of OHSS.

Results of univariate and multivariate regression analyses with the number of oocytes as a dependent variable are shown in Table 2. The independent variables were: age, height, weight, BMI, smoking status, day3 FSH, estradiol and AFC. The univariate regression analysis showed that the number of retrieved oocytes was significantly predicted by age, BMI, smoking status, d3FSH and AFC, however in the multivariate regression analysis the statistical significance was reached only for age, serum d3FSH and AFC. The model based on multiple regression analysis accounts for 30% of the variability of ovarian sensitivity (R2=0.31; R2-adjusted: 0.3). According to the model, for women of similar age, the number of retrieved oocytes will be reduced with decreasing of AFC and increasing of day 3 serum FSH. The most significant predictors of ovarian sensitivity were FSH (P=0.0001) and AFC (P<0.0001).

**Table 2:** Predictors of number of retrieved oocytes in univariate and multivariate backward regression analysis

** Variable**	**Univariate**			**Multivariate**		
	**Regresssion coefficient**	**Standard Error**	**p**	**Regresssion coefficient**	**Standard Error**	**p**
Age	−0,28116	0,05076	<0.0001	−0,08732	0,05046	0.02
FSH	−0,32630	0,06085	<0.0001	−0,22924	0,05726	0.0001
AFC	0,41664	0,03041	<0.0001	0,35517	0,03538	<0.0001
BMI	−0,002	0,00005	0.01	−0,001	0,00005	ns
Height	−0,01227	0,00358	ns	−0,01	0,003	ns
Weight	0,04202	0,02488	0.09	0,03	0,02	ns
Basal estradiol	−0,00664	0,00503	ns	−0,004	0,005	ns
Smoking status	−0.00051	0.00008	0.01	−0.0005	0.00003	ns

**Figure 1:**
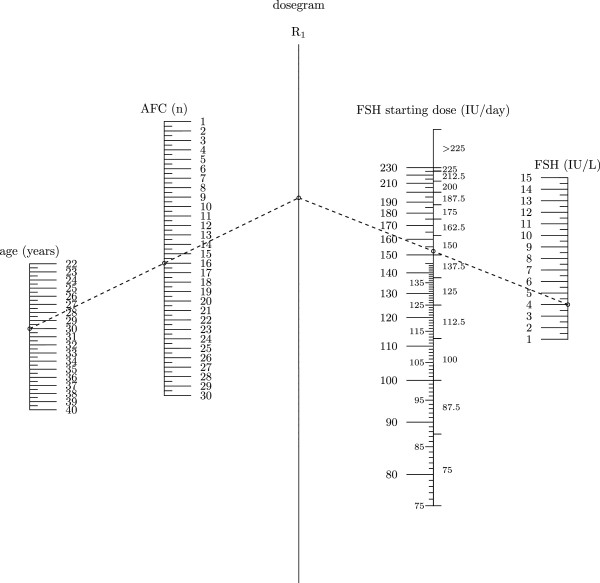
**The nomogram for the calculation of the FSH starting dose based on age, AFC and serum day 3FSH.** In the example, for a 30 year old woman with AFC=16 and d3FSH = 4IU/L, the FSH starting dose is 152 IU per day. Since the new FSH delivery system will have the dosage dial based on doses of FSH of 12.5 IU, on the right side of the FSH starting dose column, the FSH dose as selected for the delivery system is reported (150 IU /day for the example).

The model shown in Table 2 was then used to elaborate a dosage normogram which could be easily adopted in daily clinical practice (Figure 1). The FSH dosage normogram was constructed after setting the desirable number of retrieved oocytes as nine. The optimal number of oocytes was set as nine since it is the mean (and median) number of retrieved oocytes in the present study.

**Figure 2:**
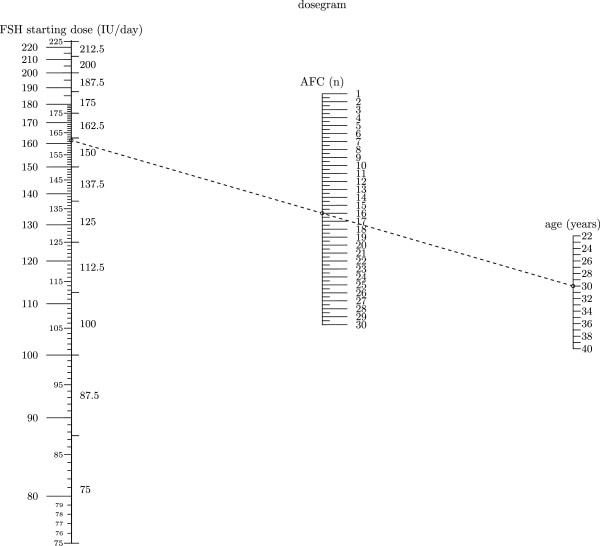
**The nomogram for the calculation of the FSH starting dose based on age and AFC.** In the example, for a 30 year old woman with AFC=16 , the FSH starting dose is 161.5 IU per day. Since the new FSH delivery system will have the dosage dial based on doses of FSH of 12.5 IU, on the right side of the FSH starting dose column, the FSH dose as selected for the delivery system is reported (150 IU /day for the example).

**Figure 3:**
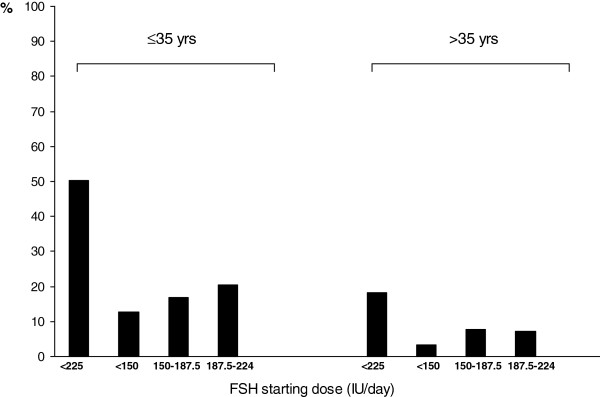
**The FSH starting dose for patients included in the study was calculated by the nomogram incorporating age, AFC and FSH.** Overall the model predicts a dose lower than 225 IU of FSH in 50.2% of patients ≤35 yrs (12.8%, 16.9% and 20.5% of patients had a predicted daily dose of <150, 150–187.5 and 187.5-225 IU, respectively). The percentage of women >35 yrs with a predicted dose lower than 225 IU was 18.1% (3.2%, 7.8% and 7.1% of women had a predicted daily dose of <150, 150–187.5 and 187.5-225 IU, respectively).

A simplified model based only on age and AFC was developed (Figure 2). Of course this model has a lower accuracy than the three-variables model (R2-adjusted: 0.25 vs 0.3, respectively). The three-variables-nomogram reported in Figure 1 was then applied on the same population on which it was calculated (n=505). Overall the model predicted a FSH starting dose lower than 225 IU in 30% of patients. Interestingly, this percentage was higher for younger patients (≤ 35 yrs: 50.2%) than for older patients (>35 yrs: 18.1%) (Figure 3).

## Discussion

Results of the present study confirm that ovarian response to a standard dose of FSH mainly depends on ovarian reserve. Indeed, in the present study the number of retrieved oocytes is significantly predicted by age and two markers of ovarian reserve namely AFC and FSH, with the last two variables being the most significant predictors.

The multiple follicular growth in cycles of assisted reproduction is effected using exogenous FSH, leading to supranormal circulating concentrations and recruitment of all follicles whose FSH sensitivity threshold is exceeded [14,15]. When exogenous FSH is administered, the number of follicles induced to grow largely depends upon the number of follicles and their FSH sensitivity [16,17]. That is the main reason why an algorithm including AFC and serum d3FSH may correctly predict the number of retrieved oocytes following ovarian stimulation.

The prediction of oocyte yield is the basis for tailoring the treatment in the daily clinical practice. Indeed the choice of the appropriate starting dose of FSH can only be secondary to an accurate prediction of oocyte yield following maximal ovarian stimulation. If the predicted oocyte yield is suggestive for excessive ovarian response the starting dose of FSH should be lower than the one leading to the maximal stimulation. For this reason, some authors examined and tested complex models based on multiple phenotypic, ultrasound derived and biochemical indexes to dictate starting doses of exogenous gonadotrophins in IVF cycles [18,19].

A first prospective study showed that the combination of age, antral follicle count (AFC), ovarian volume, Doppler ovarian score and smoking status may allow clinicians to choose the appropriate FSH dose in IVF cycles [19]. In a second study the proposed model was based on age, body mass index (BMI), serum day 3 FSH (d3FSH) and AFC [18]. Both models were then validated in successive prospective trials, demonstrating that the application of an individualized versus standard FSH dose was associated with a reduced cancellation rate for abnormal ovarian response, reduced need of adjusting the dose during treatment and increased occurrence of an adequate ovarian response [20,21]. Unfortunately both the published nomograms may not be used by clinicians in their practice since the nomogram by Popovic-Todorovic [19] was based on a Doppler score of ovarian stromal blood flow and testosterone levels, which are not commonly measured in the clinical practice, whereas the model created by Howles [18] has never been publicly disclosed.

In the present study ovarian response is significantly predicted by age, serum FSH and AFC and this model may permit the construction of an FSH dosage nomogram. The nomogram (Figure 1) was constructed after setting the desirable number of retrieved oocytes as nine. As already said, the hypothesis was that stimulating the ovaries wishing to obtain a number of oocytes close to the median in the whole population leads to a narrow distribution of ovarian responses around that value. This would imply that fewer women would exhibit an inadequate response (i.e. poor or excessive response). This methodology has been successfully used in previously published models [13,18,19]. Moreover nine is the middle point of the appropriate ovarian response when defined as the retrieval of 5 to 14 oocytes [19,22] and the middlepoint between the cut-off values usually used to define “poor response” (<4 oocytes) [23] and “excessive ovarian response” (>15) [8,24-26].

The need of individualizing doses of FSH in patients derives from the assumption that variability in the functional ovarian reserve (the pool of recruitable follicles) is very wide; therefore a standard fixed dose of FSH may not be suitable for all women [27]. Indeed several observations [19,21,22] indicate that 150 IU of FSH is the optimal dose for part of the studied patients, with others requiring higher doses of FSH in order to reach the optimal stimulation. On the other hand a large proportion of OHSS cases are preventable by reducing the FSH starting dose in women at risk of excessive ovarian response, namely those with a large pool of recruitable follicles.

Everything previously enounced clearly indicates that the starting dose of FSH in IVF cycles should be individualized. In the present study we clearly demonstrated that the FSH daily dose may be calculated on the basis of the age of patients and of two markers of ovarian reserve, namely AFC and FSH, with the last two variables being the most significant predictors. The model based on AFC resembles a previously published model based on AMH [13]. The two models have the same accuracy in predicting ovarian response to gonadotropins and this is not surprising since a linear strong relationship exists between AFC and AMH [4,7]. The strong correlation between the two markers reflects the fact that the same ovarian follicles which are seen on ultrasound secrete AMH. One relevant benefit of AFC is the possibility of its measurement at the same moment in which clinicians examine the patient hence we developed a second simplified model based only on age and AFC (Figure 2). As expected the two variables model is less accurate than the one incorporating age, FSH and AFC, but being independent of the blood sample makes it highly interesting for clinicians.

When the nomogram we developed was tested in the same population used to elaborate the model (Figure 3), it predicted a dose lower than 150 IU in 8% of patients, a dose between 150 and 187.5 IU in 11.5% of patients, or between 187.5 and 225 IU in 10.5% of patients. In women ≤ 35 years the calculated FSH starting dose was lower than 225 IU in 50.2% of patients while it was lower than 225 IU only in 18.1% of women > 35 years. Before clinicians can adopt the model into routine clinical practice, the accuracy of the model should be independently evaluated in a population different from the one on which the model was elaborated. External validation is therefore crucial to assess the generalizability of our model to other populations.

## Conclusions

We have demonstrated that ovarian response to COS depends on patient’s age and on the pool of recruitable follicles and follicular sensitivity to FSH, which may be indirectly measured using markers of ovarian reserve and sensitivity, namely AFC and serum day 3 FSH. The prediction of ovarian response to maximal stimulation is the basis for the modulation of the dose of FSH to administer to patients undergoing IVF. By modulating the FSH starting dose, clinicians may increase the number of patients with a satisfactory oocyte yield while reducing both the extremes. Of course the validation of the proposed FSH dosage nomogram in a large prospective study is needed.

## Abbreviations

AFC: Antral follicle count; IVF: In vitro fertilization; PCO: Polycystic ovaries; PID: Pelvic inflammatory disease; d3FSH: Day 3 FSH; OHSS: Ovarian hyper-stimulation syndrome; COS: Controlled ovarian stimulation.

## Competing interests

The authors declare that they have no competing interests.

## Authors’ contribution

ALM and EP prepared the data and drafted the manuscript. ALM, SG and AT performed the statistical analysis. VG, CA and GD edited the manuscript. ALM and AV were responsive for the project, data integrity and final edit of the manuscript. All authors read and approved the final manuscript.
